# Multiuse of Bar-HRM for *Ophiocordyceps sinensis* identification and authentication

**DOI:** 10.1038/s41598-018-31164-4

**Published:** 2018-08-24

**Authors:** Maslin Osathanunkul, Khukrit Osathanunkul, Sutthipan Wongwanakul, Rossarin Osathanunkul, Panagiotis Madesis

**Affiliations:** 1 Department of Biology, Faculty of Science, Chiang Mai University, Chiang Mai, 50200 Thailand; 2 Center of Excellence in Bioresources for Agriculture, Industry and Medicine, Chiang Mai University, Chiang Mai, 50200 Thailand; 3 Department of Information Technology, The International Collage, Payap University, Chiang Mai, 50000 Thailand; 4 Department of Urology, McCormick Hospital, Chiang Mai, 50000 Thailand; 5 Faculty of Economics, Chiang Mai University, Chiang Mai, 50200 Thailand; 6 Institute of Applied Biosciences, Centre for Research & Technology Hellas (CERTH), Thessaloniki, Greece

## Abstract

Bar-HRM is a hybrid method which combines DNA barcoding and High Resolution Melting analysis. It has proven to be a fast, cost-effective and reliable molecular approach for species identification and authentication. Here, three aspects of the use of Bar-HRM are focused on. First, Bar-HRM is used to discriminate between closely related *Ophiocordyceps* species. Second, identification of an unknown powder that is claimed to be *Ophiocordyceps* species using Bar-HRM. Third, authenticating the *O*. *sinensis* products sold on the market by the Bar-HRM. Results from HRM analyses with ITS primers shows that the two *Ophiocordyceps* species (*Ophiocordyceps sinensis* and *Ophiocordyceps militaris*) were easily differentiated. Also, an unknown sample was able to be identified in less time compared with using DNA barcoding alone. In addition, the substitution or adulteration of *O*. *sinensis* products sold on market was detected via Bar-HRM. The substitution or adulteration of inferior *Ophiocordyceps* species, particularly *O*. *militaris* in high price *O*. *sinensis* products has been a concern throughout Asia. Based on our results, the Bar-HRM was again proved to be a promising tool for species identification and authentication.

## Introduction

In 2003, DNA barcoding was proposed^1^ as a molecular approach for species identification and has become popular since then. DNA barcodes have been successfully used in animal species identification. The region of the mitochondrial cytochrome *c* oxidase subunit 1 (COI) has been successfully used as the animal barcode, but it is difficult to amplify in fungi and often includes large introns, and it can be insufficiently variable. The internal transcribed spacer (ITS) region has the highest probability of successful identification for the broadest range of fungi^2,3^. Recently, DNA barcoding approach has been applied to use for detection of adulteration in food and herbal products with success (e.g.^4–6^). However, some limitations of the method mean it is not fully practical in some developing countries like Thailand. The main drawbacks are likely to be that it is time-consuming and its high costs due to outsourced sequencing. To overcome those limitations, DNA barcoding has been applied to be used in combination with High Resolution Melting analysis, so-called Bar-HRM. Bar-HRM is a sequencing free method using fluorescent dye for detection of double-stranded DNA in real-time PCR increasing temperature during the process leads to denaturation of double-stranded DNA into single-stranded DNA and melting temperature (T_m_) is measured. The Bar-HRM analysis is not only rapid, cheap (in long term and large scale investigation), and feasible for accurate species discrimination in plants^7–10^. It was also was proven to be a good compromise for counterfeiting herbal products and adulteration detection^11–17^. As a consequence, the Bar-HRM is one of many promising techniques not only for species identification/discrimination but also for counterfeit/adulterant detection in commercial products sold on the market. Here, the *Ophiocordyceps* species is the main focus.

A parasitic fungus known as *Ophiocordyceps* has a long history of use in Asian and is commonly used as a herbal medicine and health supplement. Based on numerous studies, *Ophiocordyceps* was found to possess anti-cancer, anti-proliferative, and anti-fibrotic^18^, anti-bacterial^19^, anti-oxidation^20^, anti-fatigue, anti-aging and neuroprotective effects^21^.

In Thailand, two major *Ophiocordyceps* species are currently popular, *Ophiocordyceps sinensis* and *Ophiocordyceps militaris*. Although *O*. *militaris* is closely related to *O*. *sinensis* and there are some overlap medicinal uses of these two, *O*. *sinensis* is rare and expensive. *O*. *sinensis* can only grow slowly in high-altitude habitats and cannot be produced by aseptic mycelia cultivation in Thailand. In contrast, *O*. *militaris* is now commonly cultivated and thus less expensive than that *O*. *sinensis*. In addition, *O*. *militaris* has been used to form adulterants which are found in products sold in Asian markets not just Thailand^22^.

Bar-HRM therefore seems to be a good tool to be used for discrimination between the two closely *Ophiocordyceps* species. In addition, it is also difficult to identify the species of processed or powdered products such as capsule and tablet. Bar-HRM has been proven to be an efficient and reliable method in doing such difficult task. Here, three cases of species identification and/or discrimination of *Ophiocordyceps* are presented to show that the Bar-HRM is one promising approach in aiding species identification.

## Results and Discussion

### Literature Review

**Figure 1:**
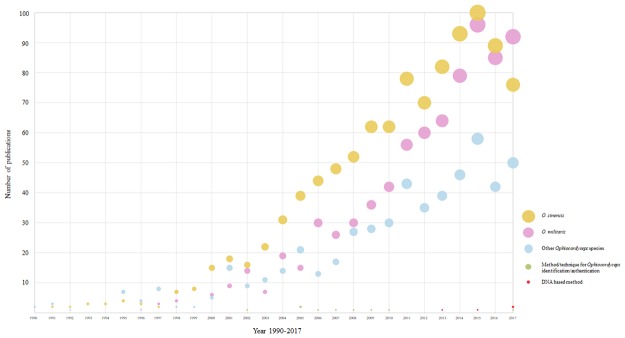
Cumulative number of *Ophiocordyceps* studies over time (1990–2017). Two main *Ophiocordyceps* species (*Ophiocordyceps sinensis* and *Ophiocordyceps militaris*) were focused on. Also the number of publications on a particular research field, method/technique for identification/authentication of *Ophiocordyceps* were shown.

Our initial literature search returned 2,181 potentially relevant publications and a refined search allowed us to narrow this to 1,846 relevant publications which focussed on only the two popular species included *Ophiocordyceps sinensis* and *Ophiocordyceps militaris* (Fig. 1). Most publications are related to one of three general categories: Pharmacology Pharmacy, Biotechnology Pharmacy or Food Science Technology. Citations indicating ideas across these three general categories were mainly to do with the medicinal properties of the *Ophiocordyceps* species. We then furthered our search for publications dealing with ‘method or technique’ for identification or authentication. The search returned only 75 publications which a review of abstracts allowed us to narrow to 12 publications reporting the use of DNA. Only 3 out of 12 articles were using DNA barcodes for *Ophiocordyceps* species identification and/or authentication^19,23,24^, whilst the rest used DNA in aiding taxonomy or nomenclature. What can be clearly seen in Fig. 1 is the continual growth of studies related to *Ophiocordyceps*, however only 3 from total of 2,181 publications which accounts for approximately 0.14% of that identification and/or authentication of *Ophiocordyceps* based on DNA. Few techniques have been used to distinguish between *Ophiocordyceps* species such as capillary electrophoresis^25^, high performance liquid chromatography (HPLC)^27,27^ and microscopic examination^28^. Although, extensive research has been carried out on methods used for quality control of *O*. *sinensis* (see review e.g.^22,29,30^), no single study exists reporting the use of DNA barcoding coupled with High Resolution Melting analysis (Bar-HRM).

Bar-HRM was proved to be a powerful tool for species identification that is capable not only to identify but also to quantitatively detect adulterants. The Bar-HRM analysis is not only rapid, cheap (in long term and large scale investigation), but it is also feasible for accurately species discrimination in various species. Thus, Bar-HRM holds a great potential to be applicable in *Ophiocordyceps* identification and/or authentication. Research on the *Ophiocordyceps* identification and/or authentication subject has been mostly restricted to limited comparisons of Pharmacology. Our study could be one to fill in this gap.

### Real-time PCR for high resolution melting (HRM) analyses

Three aspects uses of Bar-HRM to identify or authenticate *O*. *sinensis* were evaluated here. Firstly, Bar-HRM was used in aiding species discriminating of two related close *Ophiocordyceps* species. Secondly, Bar-HRM was applied to identify unknown powder. Lastly, we authenticated *Ophiocordyceps* commercial product sold on the markets by Bar-HRM.

### Experiment 1: Differentiation of *Ophiocordyceps* raw materials

**Figure 2:**
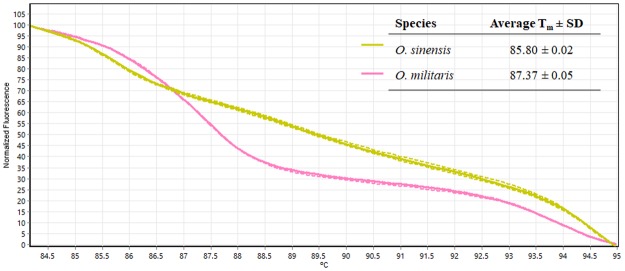
The normalised plot shows the differentiation of melting temperature (T_m_) of each ITS amplicon from each *Ophiocordyceps* species, generated by high resolution melting (HRM) analysis.

The feasibility of *Ophiocordyceps* species discrimination in Bar-HRM technique was examined with two *Ophiocordyceps* species included *O*. *sinensis* and *O*. *militaris*. Raw materials of the two *Ophiocordyceps* species were obtained and tested. Genomic DNA was extracted from the samples and taken to be used in HRM analysis with ITS primer pairs. HRM analysis was performed in triplicate on each of the tested species to establish the T_m_. The shapes of the melting curves were analysed using Rotor-Gene Q Series Software (v. 2.3.1) to distinguish between the different *Ophiocordyceps* species. The ITS primer set yielded amplicons of the expected size, approximately 315 base-pairs long. Figure 2 depicts the analysis by means of conventional derivative plots, which show the T_m_ value for the ITS fragment from each species. The melting temperatures of the *O*. *sinensis* were 85.80 ± 0.02 °C, and *O*. *militaris* was 87.37 ± 0.05 °O. The two different *Ophiocordyceps* species could be distinguished by using HRM analysis. A distinct melting curve was generated for each *Ophiocordyceps* species presenting one inflection point (Fig. 2). It is indicated that the two *Ophiocordyceps* species could be discriminated with Bar-HRM.

### Experiment 2: Species identification of unknown powder sample

**Figure 3:**
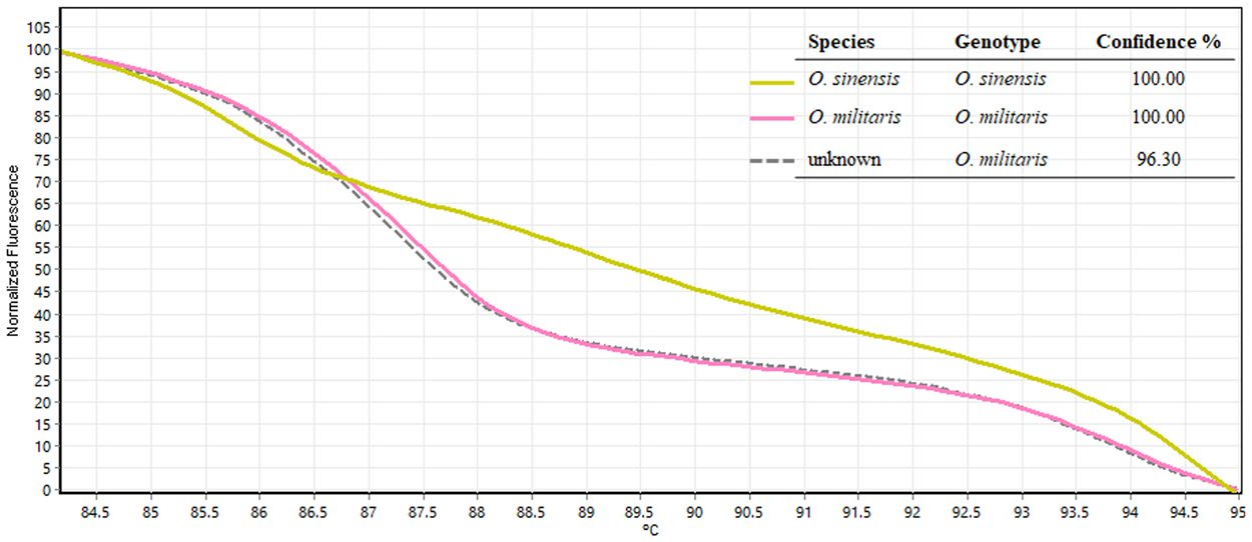
Melting curve profiles of amplicons obtained from ITS primers from an unknown sample and the two reference *Ophiocordyceps* (*O*. *sinensis* and *O*. *militaris*).

We received a short notice request from Chiang Mai International Airport’s (Airports of Thailand Public Company Limited: AOT) officers. They wanted us to confirm the species of powder sample carried by a passenger which claimed to be *O*. *militaris*. A quick HRM analysis was then performed right after obtaining the sample. Two *Ophiocordyceps* specimens (*O*. *sinensis* and *O*. *militaris*) tested in Experment 1 were used as reference species for the analysis. As can be seen in Fig. 3, a melting curve of an unknown sample was similar to the *O*. *militaris*’ curve with 96.30% confidence. After about 2.30 hours, we could confirm that the powder sample contained *O*. *militaris* as claimed (we also double checked our results with DNA sequencing of the sample, the sequence came two weeks later, sequencing results are shown in Supplementary Data 1). The analysis was carried out in three replicates. Here, it is shown that Bar-HRM is one simple, rapid and efficient method for *Ophiocordyceps* identification.

### Experiment 3: Authentication of Ophiocordyceps commercial products

**Figure 4:**
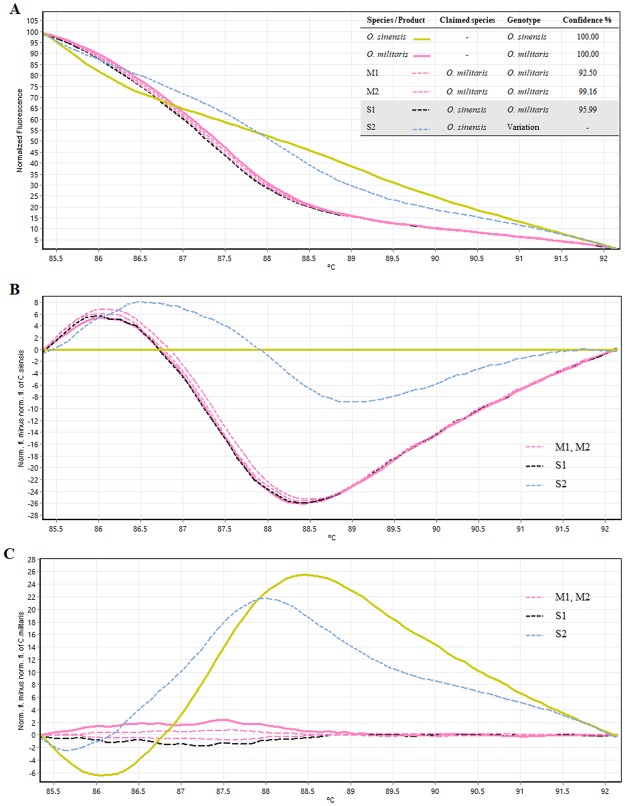
Melting curve profiles of all tested products and the two *Ophiocordyceps* species which are *O*. *sinensis* and *O*. *militaris*. (**A**) Normalised melting curve, (**B**) Difference melting curves setting *O*. *sinensis* as a reference and (**C**) Difference melting curves setting *O*. *militaris* as a reference.

The Bar-HRM was performed to authenticate the commercial *Ophiocordyceps* products sold on markets. It is undeniable that substituting or adulterating of inferior *Ophiocordyceps* species was reported througout the market in Asia. Here, four *Ophiocordyceps* products were tested. Two tested products were claimed as *O*. *sinensis* (S1 and S2) and other two were claimed as *O*. *militaris* (M1 and M2). The examination of the HRM difference curve of both samples revealed that the M1 and M2 produced curves which were the same as *O*. *militaris*’s, with a 90% confidence interval, suggesting that the products contain *O*. *militaris* (Fig. 4A). Thus, the two *O*. *militaris* tested products (M1 and M2) showed no substitution or adulteration. In contrast, results from HRM analysis indicated the substitution/adulteration/contamination of *O*. *militaris* in products claimed as *O*. *sinensis* (S1 and S2). The melting curve of S1 was distinctly different from the *O*. *sinensis*’s and nearly identical to the *O*. *militaris*’s (Fig. 4A). Therefore, the species contained in S1 is likely to be *O*. *militaris* not the *O*. *sinensis* as indicated in the package. In addition, the melting profile of S2 was not similar to any of the two *Ophiocordyceps* reference curves in which this could be result from mixing of the two species in the product or other species (Fig. 4A). Figures 4B,C represent difference curves of the test products and reference species. *O*. *sinensis* was set as a reference species in Fig. 4B and it is clearly shown that none of the tested products (M1-M2 ans S1-S2) produced same melting curves as the *O*. *sinensis*. Also, when the *O*. *militaris* was set as as a reference species in Fig. 4C, it was found that melting curves of the three tested products (M1-M2 and S1) share similarity with the reference. Both of the *O*. *militaris* products tested here were likely to contain the *O*. *militaris* as claimed. However, both *O*. *sinensis* products were found to be substitued and/or adulteranted with *O*. *militaris*. Our results are similar to those reported by Li^22^. As the results very clearly demonstrate, it is important to have a standard or strict quality control of *O*. *sinensis* products.

In Thailand, *Ophiocordyceps* products are commonly known as ‘Tang Chao’ and are rarely specifically indicated as *Ophiocordyceps* species in advertisement. As *O*. *sinensis* and *O*. *militaris* share the same name ‘Tang Chao’, several consumers bought products without realising the species of *Ophiocordyceps* in the products. Such buyer or consumer behaviour could lead to fraudulent, intentional substitution of inferior *Ophiocordyceps* species in a product for economic gain.

## Conclusion

Most studies in the field of *Ophiocordyceps* have mainly focused on pharmacology. Few publications about method or technique use in species identification can be found, although this is one of the most important related subjects. Either basic or applied fields such as taxonomy, nomenclature, food science and industry would benefit from having a reliable technique for species identification. Here, the Bar-HRM was proved to be efficient and rapid method for *Ophiocordyceps* species identification and authentication. In all three aspects of using Bar-HRM to identify or discriminate *Ophiocordyceps* species in (1) raw materials, (2) unknown sample, and (3) commercial products was successful.

## Methods

### Literature Review

On 4 April 2018, we conducted a Thomson Reuters Web of Science search using the following search term ‘Ophiocordyceps’. From the search results, we identified publications that focus on ‘sinensis’ or ‘militaris’ species. We then defined the search results into publication year from 1990–2017. Based on articles only, we further refined our search with the term ‘method’ OR ‘technique’ AND ‘identification’ OR ‘authentication’.

### Experiment 1: Differentiation of Ophiocordyceps raw materials

DNA was extracted with the Nucleospin Plant II kit (Macherey-Nagel, Germany) following the manufacturer’s instructions. DNA concentrations were adjusted to a final concentration of 20 ng/μL. The DNA was stored at −20 °C for further use. DNA of the two closely related *Ophiocordyceps* species were then used for Bar-HRM analysis. To determine the characteristic melting temperature (T_m_) for each sample that could be used to distinguish the two different species, DNA amplification using real-time PCR was performed using the Rotor-Gene Q 5plex HRM system (Qiagen, Germany). The reaction mixture for the real-time PCR and HRM analysis consisted of a total volume of 10 µl, containing 5 µl of MeltDoctor HRM Master Mix (Applied Biosystems, USA), 0.2 µl of 10 mM forward primer, 0.2 µl of 10 mM reverse primer, 1 µl of 20 ng DNA and 3.6 µl of ddH_2_O. The nucleotide of forward and reverse primers^31^ were ITS5 5′-GGAAGTAAAAGTCGTAACAAGG-3′ and ITS2 5′-GCTGCGTTCTTCATCGATGC-3′. Fluorescence dye was used to monitor both the accumulation of the amplified product and the high-resolution melting process in order to derive the T_m_ value during PCR. The reaction conditions were as follows; an initial denaturing step at 95 °C for 5 min followed by 40 cycles of 95 °C for 30 s, 57 °C for 30 s and 72 °C for 20 s. Melting curves were generated after the last extension step. The temperature for the HRM analysis was increased from 60 to 95 °C at 0.1 °C/s.

### Experiment 2: Species identification of unknown powder sample

We received a request from Chiang Mai International Airport (Airports of Thailand Public Company Limited: AOT) to confirm the species contain in powder sample taken from a passenger who carried the sample and claimed that it is the *Ophiocordyceps*. DNA extraction, DNA amplification using real-time PCR were performed as described in Experiment 1.

### Experiment 3: Authentication of Ophiocordyceps commercial products

Two commercial products claimed to be *O*. *sinensis* (S1 and S2) and two claimed to be *O*. *militaris* (M1 and M2) were purchased and tested. DNA extraction, DNA amplification using real-time PCR were performed as described in Experiment 1.

## Electronic supplementary material

Supplementary Data 1.
