# Supplementary material for: Multiuse of Bar-HRM for Ophiocordyceps sinensis identification and authentication
Source: Sci Rep. 2018 Aug 24;8:12770. doi: 10.1038/s41598-018-31164-4 (PMC6109059; doi:10.1038/s41598-018-31164-4)
Supplement: Supplementary file 1 — Supplementary Data 1. [file 41598_2018_31164_MOESM1_ESM.pdf]

Multiuse of Bar-HRM for *Ophiocordyceps sinensis* identification and authentication

Maslin Osathanunkul[1,2], Khukrit Osathanunkul[3], Sutthipan Wongwanakul[4], Rossarin Osathanunkul[5], Panagiotis Madesis[6]

[1]Department of Biology, Faculty of Science, Chiang Mai University, Chiang Mai, Thailand

[2]Center of Excellence in Bioresources for Agriculture, Industry and Medicine, Chiang Mai University

[3]Department of Information Technology, The International Collage, Payap University, Chiang Mai, Thailand

[4]Department of Urologist, McCormick Hospital, Chiang Mai, 50000, Thailand

[5]Faculty of Economics, Chiang Mai University, Chiang Mai, 50200, Thailand

[6]Institute of Applied Biosciences, Centre for Research & Technology Hellas (CERTH), Thessaloniki, Greece

*Corresponding author: Maslin Osathanunkul, Department of Biology, Faculty of Science, Chiang Mai University, Chiang Mai 50200, Thailand.

E-mail: omaslin@gmail.com. Phone: +66 53 943348. Fax: +66 53 892259.

Supplementary Data 1. Sequencing results confirming of each experiment

> EXP1_ O. sinensis

CATTATCGAGTCACCACTCCCAAACCCCCTGCGAACACCACAGCAGTTGCCTCGGCGGGACCGCCCCGGCGCCCCAGGGCCCGGACCAGGGCGC
CCGCCGGAGGACCCCCAGACCCTCCTGTCGCAGTGGCATCTCTCAGTCAAGAAGCAAGCAAATGAATCAAAACTTTCAACAACGGATCTCTTGG
TTCTGGCATCGATGAAGAACGCAGCGAAATGCGATAAGTAATGTGAATTGCAGAATTCAGTGAACCATCGAATCTTTGAACGCACATTGCGCCC
GCCAGCACTCTGGCGGGCATGCCTGTCCGAGCGTCATCTCAACCCTCGAGCCCCCCGCCTCGCGGCGGCGGGGCCCGGCCTTGGGGGTCACGGC
CCCGCGCCGCCCCCTAAACGCAGTGGCGACCCCGCCGCGGCTCCCCTGCGCAGTAGCTCGCTGAGAACCTCGCACCGGGAGCGCGGAGGCGGTC
ACGCCGTGAAACCACCACACCCTCCAGTTGACCTCGGATCAGGTAGGGATACCCGCTGAACTTAAGCATA

>EXP1_ O. militaris

CGTTCAGAGTTGGGCGTTTTACGGCGTGGCCACGTCGGGTTCCCGGTGCGAGTTGGAGTACTACGCAGAGGTCGCCGCGGACGGGCCGCCACTT
CATTTCGGGGGCGGCGGTGTGCTGCCGGTCCCCAACGCCGACATCCCCCAGGGGACGTCGAGGGTTGAAATGACGCTCGAACAGGCATGCCCGC
CAGAATGCTGGCGGGCGCAATGTGCGTTCAAAGATTCGATGATTCACTGAATTCTGCAATTCACATTACTTATCGCATTTCGCTGCGTTCTTCATC
GATGCCAGAGCCAAGAGATCCGTTGTTGAAAGTTTTGATTCATTTGTTTTGCCTTGCGGCGGATTCAGAAAAACTGGTAGATACAGTGTTTGGGG
CCCCCGACGGCCGCCGCCCAGGCCCGCGTCCAGGCGCTGGGCGAGTCCGCCGAAGCAACGATAGGTATGTTCACAAAGGGTTGGGAGTTGGAA
AACTCGTTAATG

>EXP2_unknown

CGTTCAGAGTTGGGCGTTTTACGGCGTGGCCACGTCGGGTTCCCGGTGCGAGTTGGAGTACTACGCAGAGGTCGCCGCGGACGGGCCGCCACTT
CATTTCGGGGGCGGCGGTGTGCTGCCGGTCCCCAACGCCGACATCCCCCAGGGGACGTCGAGGGTTGAAATGACGCTCGAACAGGCATGCCCGC
CAGAATGCTGGCGGGCGCAATGTGCGTTCAAAGATTCGATGATTCACTGAATTCTGCAATTCACATTACTTATCGCATTTCGCTGCGTTCTTCATC
GATGCCAGAGCCAAGAGATCCGTTGTTGAAAGTTTTGATTCATTTGTTTTGCCTTGCGGCGGATTCAGAAAAACTGGTAGATACAGTGTTTGGGG
CCCCCGACGGCCGCCGCCCAGGCCCGCGTCCAGGCGCTGGGCGAGTCCGCCGAAGCAACGATAGGTATGTTCACAAAGGGTTGGGAGTTGGAA
AACTCGTTAATG

>EXP3_M1

CGTTCAGAGTTGGGCGTTTTACGGCGTGGCCACGTCGGGTTCCCGGTGCGAGTTGGAGTACTACGCAGAGGTCGCCGCGGACGGGCCGCCACTT
CATTTCGGGGGCGGCGGTGTGCTGCCGGTCCCCAACGCCGACATCCCCCAGGGGACGTCGAGGGTTGAAATGACGCTCGAACAGGCATGCCCGC
CAGAATGCTGGCGGGCGCAATGTGCGTTCAAAGATTCGATGATTCACTGAATTCTGCAATTCACATTACTTATCGCATTTCGCTGCGTTCTTCATC
GATGCCAGAGCCAAGAGATCCGTTGTTGAAAGTTTTGATTCATTTGTTTTGCCTTGCGGCGGATTCAGAAAAACTGGTAGATACAGTGTTTGGGG
CCCCCGACGGCCGCCGCCCAGGCCCGCGTCCAGGCGCTGGGCGAGTCCGCCGAAGCAACGATAGGTATGTTCACAAAGGGTTGGGAGTTGGAA
AACTCGTTAATG

>EXP3_M2

CGTTCAGAGTTGGGCGTTTTACGGCGTGGCCACGTCGGGTTCCCGGTGCGAGTTGGAGTACTACGCAGAGGTCGCCGCGGACGGGCCGCCACTT
CATTTCGGGGGCGGCGGTGTGCTGCCGGTCCCCAACGCCGACATCCCCCAGGGGACGTCGAGGGTTGAAATGACGCTCGAACAGGCATGCCCGC
CAGAATGCTGGCGGGCGCAATGTGCGTTCAAAGATTCGATGATTCACTGAATTCTGCAATTCACATTACTTATCGCATTTCGCTGCGTTCTTCATC
GATGCCAGAGCCAAGAGATCCGTTGTTGAAAGTTTTGATTCATTTGTTTTGCCTTGCGGCGGATTCAGAAAAACTGGTAGATACAGTGTTTGGGG
CCCCCGACGGCCGCCGCCCAGGCCCGCGTCCAGGCGCTGGGCGAGTCCGCCGAAGCAACGATAGGTATGTTCACAAAGGGTTGGGAGTTGGAA
AACTCGTTAATG

>EXP3_S1

CGTTCAGAGTTGGGCGTTTTACGGCGTGGCCACGTCGGGTTCCCGGTGCGAGTTGGAGTACTACGCAGAGGTCGCCGCGGACGGGCCGCCACTT
CATTTCGGGGGCGGCGGTGTGCTGCCGGTCCCCAACGCCGACATCCCCCAGGGGACGTCGAGGGTTGAAATGACGCTCGAACAGGCATGCCCGC
CAGAATGCTGGCGGGCGCAATGTGCGTTCAAAGATTCGATGATTCACTGAATTCTGCAATTCACATTACTTATCGCATTTCGCTGCGTTCTTCATC
GATGCCAGAGCCAAGAGATCCGTTGTTGAAAGTTTTGATTCATTTGTTTTGCCTTGCGGCGGATTCAGAAAAACTGGTAGATACAGTGTTTGGGG
CCCCCGACGGCCGCCGCCCAGGCCCGCGTCCAGGCGCTGGGCGAGTCCGCCGAAGCAACGATAGGTATGTTCACAAAGGGTTGGGAGTTGGAA
AACTCGTTA

>EXP3_S2(1)

CGTCGGGTTCCCGGTGCGAGTTGGAGTACTACGCAGAGGTCGCCGCGGACGGGCCGCCACTTCATTTCGGGGGCGGCGGTGTGCTGCCGGTCCC
CAACGCCGACATCCCCCAGGGGACGTCGAGGGTTGAAATGACGCTCGAACAGGCATGCCCGCCAGAATGCTGGCGGGCGCAATGTGCGTTCAA
AGATTCGATGATTCACTGAATTCTGCAATTCACATTACTTATCGCATTTCGCTGCGTTCTTCATCGATGCCAGAGCCAAGAGATCCGTTGTTGAAA
GTTTTGATTCATTTGTTTTGCCTTGCGGCGGATTCAGAAAAACTGGTAGATACAGTGTTTGGGGCCCCCGACGGCCGCCGCCCAGGCCCGCGTCC
AGGCGCTGGGCGAGTCCGCCGA

> EXP3_S2(5)

TCCCCCAGGGGACGTCGAGGGTTGAAATGACGCTCGAACAGGCATGCCCGCCAGAATGCTGGCGGGCGCAATGTGCGTTCAAAGATTCGATGAT
TCACTGAATTCTGCAATTCACATTACTTATCGCATTTCGCTGCGTTCTTCATCGATGCCAGAGCCAAGAGATCCGTTGTTGAAAGTTTTGATTCAT
TTGTTTTGCCTTGCGGCGGATTCAGAAAAACTGGTAGATACAGTGTTTGGGGCCCCCG

> EXP3_S2(6)

CCGGTGCGAGTTGGAGTACTACGCAGAGGTCGCCGCGGACGGGCCGCCACTTCATTTCGGGGGCGGCGGTGTGCTGCCGGTCCCCAACGCCGAC
ATCCCCCAGGGGACGTCGAGGGTTGAAATGACGCTCGAACAGGCATGCCCGCCAGAATGCTGGCGGGCGCAATGTGCGTTCAAAGATTCGATGA
TTCACTGAATTCTGCAATTCACATTACTTATCGCATTTCGCTGCGTTCTTCATCGATGCCAGAGCCAAGAGATCCGTTGTTGAAAGTTTTGATTCA
TTTGTTTTGCCTTGCGGCGGATTCAGAAAAACTGGTAGATACAGTGTTTGGGGCCCCCGACGGCCGCCGCCCAGGCCCGCGTCCAGGCGCTGGGC
GA
